# X-ray Imaging of Alloy Solidification: Crystal Formation, Growth, Instability and Defects

**DOI:** 10.3390/ma15041319

**Published:** 2022-02-10

**Authors:** Shikang Feng, Enzo Liotti, Patrick S. Grant

**Affiliations:** Department of Materials, University of Oxford, Oxford OX1 3PH, UK; enzo.liotti@materials.ox.ac.uk (E.L.); patrick.grant@materials.ox.ac.uk (P.S.G.)

**Keywords:** in situ X-ray imaging, solute suppressed nucleation, twin plane re-entrant growth, defect formation, additive manufacturing

## Abstract

Synchrotron and laboratory-based X-ray imaging techniques have been increasingly used for in situ investigations of alloy solidification and other metal processes. Several reviews have been published in recent years that have focused on the development of in situ X-ray imaging techniques for metal solidification studies. Instead, this work provides a comprehensive review of knowledge provided by in situ X-ray imaging for improved understanding of solidification theories and emerging metal processing technologies. We first review insights related to crystal nucleation and growth mechanisms gained by in situ X-ray imaging, including solute suppressed nucleation theory of α-Al and intermetallic compound crystals, dendritic growth of α-Al and the twin plane re-entrant growth mechanism of faceted Fe-rich intermetallics. Second, we discuss the contribution of in situ X-ray studies in understanding microstructural instability, including dendrite fragmentation induced by solute-driven, dendrite root re-melting, instability of a planar solid/liquid interface, the cellular-to-dendritic transition and the columnar-to-equiaxed transition. Third, we review investigations of defect formation mechanisms during near-equilibrium solidification, including porosity and hot tear formation, and the associated liquid metal flow. Then, we discuss how X-ray imaging is being applied to the understanding and development of emerging metal processes that operate further from equilibrium, such as additive manufacturing. Finally, the outlook for future research opportunities and challenges is presented.

## 1. Introduction

Solidification is involved in the manufacture of a large variety of engineering alloy products, ranging from direct chill (DC) cast billets to additively manufactured structural components, during which the cooling rate spans a wide range from <1 K s${}^{-1}$ to 10${}^{6}$ K s${}^{-1}$ [1,2,3]. The mechanical properties of the alloy products depend critically on the microstructure developed during solidification, including the grain size and its morphology, the size and distribution of secondary, minority phases and any microstructural defects, which are frequently difficult or not cost-effective to manipulate by down-stream, solid-state processing. Understanding solidification is therefore of importance for controlling the final alloy microstructure and mechanical properties, and it plays a crucial role in both new alloy development and optimisation of processing routes.

For nearly a century, studies of alloy solidification and other high-temperature phase transformations have been performed in a postmortem, destructive way via a combination of optical or electron microscopy (EM), chemical analysis, X-ray diffraction, calorimetry, etc. With the advent of high-energy, high-brilliance synchrotrons, and more recently the development of better laboratory-based X-ray sources together with more efficient X-ray detectors, X-ray imaging techniques such as radiography and tomography, and X-ray diffraction have been used increasingly to investigate solidification processes in real time.

Several review articles have been published in recent years on the application of in situ X-ray techniques to metal solidification [4,5,6,7,8]. For example, Y.B. Wang et al. [4] and later Y.N. Wang et al. [8] reviewed X-ray imaging of dendritic growth: Y.B. Wang et al. provided an overview of the in situ observation of different dendritic morphologies in Mg, Sn, and Al alloys [4], while Y.N. Wang et al. focused on Mg alloys and reviewed the evolution of dendrite arm orientations and growth directions as a function of alloy composition [8]. Karagadde et al. [7] and Peng et al. [6] reviewed the development of X-ray imaging techniques, especially tomography-based techniques, with Karagadde et al. also presenting advances in the development of specialised solidification rigs/environment cells. Feng et al. presented a review on in situ chemical mapping, covering experimental arrangements, their capability for capturing single or multiple elements, and the development of X-ray detectors [5].

Unlike the aforementioned works that largely focused on X-ray imaging techniques, the current work instead presents a comprehensive review of knowledge provided by in situ X-ray imaging, for better understanding of solidification theories. Thanks to the high spatial (sub-micron) and temporal (microseconds) resolution offered by third-generation synchrotron sources, significant progress has been made in the understanding, validation and development of theories and models for near-equilibrium solidification, including solute suppressed nucleation (SSN) of both primary solid-solution α-Al [9,10,11,12,13,14] and secondary ordered intermetallics [15,16], dendritic growth of α-Al [17,18,19,20,21,22] and faceted, twin plane re-entrant (TPRE) growth of Fe-rich intermetallics [23,24,25,26], crystal fragmentation [27,28,29,30,31,32], morphological transition [33,34,35,36,37,38,39,40] and defect formation [41,42,43,44,45,46,47]. The focus of solidification research on Al alloys derives from the relatively easy-to-achieve melting temperatures of around 660 ${}^{\circ }C$, and excellent absorption contrast between Al and typical alloying elements such as Cu and Zn. Further, the high temporal resolution now afforded also allows new insights to be gained on transient phenomena during non-equilibrium, rapid solidification processes, such as the effect of processing parameters on melt pool morphology and melt pool defects in additive manufacturing (AM) [48,49,50,51,52,53,54], and has also made a contribution to the development and validation of new AM processes [55,56].

## 2. Studying Alloy Solidification Using X-rays

**Figure 1:**
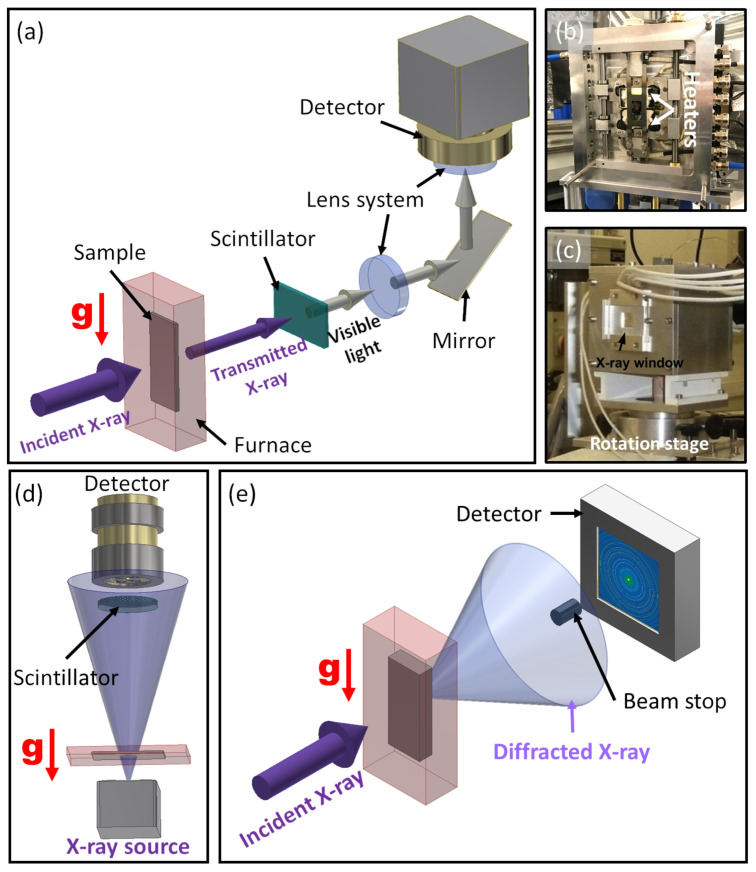
(**a**) Schematic of the experimental set-up for synchrotron X-ray radiography of solidification, where the sample is solidified vertically [5]. (**b**) An example of a Bridgman furnace for radiography experiments. (**c**) An example of a furnace for tomography experiments [60]. (**d**) Schematic of a typical experimental configuration for laboratory-based X-ray radiography of horizontal solidification (i.e., with gravity perpendicular to the sample surface). (**e**) Schematic of a typical experimental set-up for synchrotron X-ray diffraction.

In X-ray imaging, such as radiography and tomography, the X-ray beam transmitted through the sample is recorded as an intensity image by a 2D detector. Pixel intensities are inversely proportional to the X-ray absorption as the beam transits the sample. Figure 1a presents a schematic of a typical set-up for synchrotron X-ray radiography of solidification experiments, consisting of an incident X-ray beam, a static foil sample (150 to 300 μm in thickness) mounted vertically (i.e., there are gravity effects in the imaging field of view) in a Bridgman-type furnace with a controlled atmosphere (Ar or N${}_{2}$), a scintillator that converts X-rays into visible light, mirrors and objective lenses that control the imaging magnification and size of field of view and a charge-coupled device (CCD) or complementary metal oxide semiconductor (CMOS) detector [27,57]. An example of a Bridgman-type furnace for radiographic experiments is shown in Figure 1b. In this example, the furnace comprises two plate electric resistance heaters, the temperatures of which are controlled independently by a PID algorithm and monitored by K-type thermocouples.

X-ray tomography in general follows the same concept as radiography, but uses a 3D cylindrical sample (normally 1 to 3 mm in diameter) mounted on a rotating stage. An example of a furnace used for tomography is shown in Figure 1c. 2D projections recorded from the rotating, solidifying sample are reconstructed to retrieve time-resolved 3D information, provided that the sample microstructure is not subject to significant change during each rotation. A detailed review of X-ray tomography techniques including a description of tomograph reconstruction and data processing can be found in [58]. Comparing radiography and tomography, the static foil sample and the relatively simple arrangement of radiography allows experiments to be performed at a higher cooling rate (up to 30 K s${}^{-1}$ for Bridgman solidification), with a faster acquisition speed, and therefore facilitates studies of more dynamic processes such as fluid flow during dendritic solidification and melt pool behaviour in AM [50,59].

As an alternative to synchrotron X-ray imaging, recent advances in micro-focus X-ray sources and the development of more efficient scintillators and detectors have allowed in situ imaging of alloy solidification in laboratories [61]. Figure 1d presents a schematic of a typical configuration for laboratory-based X-ray radiography. Although the spatio-temporal resolution is not as good as that provided by synchrotron X-rays, the laboratory apparatus is convenient and allows samples to be solidified horizontally (with the gravity perpendicular to the imaging field of view), which offers the advantage of significantly reduced liquid convection, similar to micro-gravity conditions [62], generally not available in synchrotrons. A more detailed description of the arrangements for laboratory-based X-ray imaging can be found in [11,15,18,61,62,63,64].

Although the current review focuses principally on in situ X-ray imaging studies, it is worth noting that in situ X-ray diffraction is also increasingly applied to solidification studies, including in tandem with imaging [65,66,67,68]. Synchrotron X-ray diffraction provides information on structural evolution throughout solidification of both single-phase and multi-phase alloys, and is particularly helpful when trying to understand non-equilibrium phase transformations such as the identification of intermediate, metastable phases [65]. Compared with absorption imaging, in situ X-ray diffraction can also facilitate investigation of stresses and strains associated with solidification [66,67]. Figure 1e presents an example of a typical synchrotron X-ray diffraction set-up. Diffracted X-rays in the forward direction are recorded by a 2D amorphous silicon detector, whereas the transmitted beam is blocked by a Pb or W beam stop to avoid saturating the detector. Taking advantage of the high-energy, high-flux third-generation synchrotrons such as the beamline 11-ID-C at Advanced Photon Source (APS), Argonne National Laboratory that produces a 115 keV X-ray beam at a flux of $10^{11}$ photons per second, bulk metal samples centimetres in thickness can be studied in diffraction mode at an acquisition rate of up to 15 Hz [69,70,71].

## 3. Crystal Nucleation and Growth

When an alloy melt is cooled below its equilibrium liquidus, phase transformations from liquid to solid are initiated with the nucleation/formation of solid crystals, which is then followed by crystal growth. Controlling the crystal formation and growth plays a critical role in controlling the final grain size, morphology and distribution of cast products and therefore their mechanical performance. For example, in the Al industry, enhancement of α-Al grain nucleation by the addition of micro-scale, insoluble grain refiner particles such as TiB${}_{2}$ and TiC results in a finer, equiaxed grain structure, and better yield strength and toughness [72]. For the past century, significant effort has been spent on understanding crystal nucleation and growth, and insightful and robust nucleation and growth theories have been developed [73,74,75,76,77,78,79,80,81,82,83,84,85,86,87,88]. Since the 1990s, and particularly during the past decade, as pioneered by Mathiesen et al. [89,90], there has been a surge in the number of in situ studies of crystal formation and growth, mostly using X-ray absorption imaging, i.e., radiography [10,11,12,13,14,15,16,18,24,26,27,29,62,63,64,91,92,93] and tomography [21,32,35,94,95,96,97,98,99,100,101,102,103,104,105].

### 3.1. Crystal Nucleation

**Figure 2:**
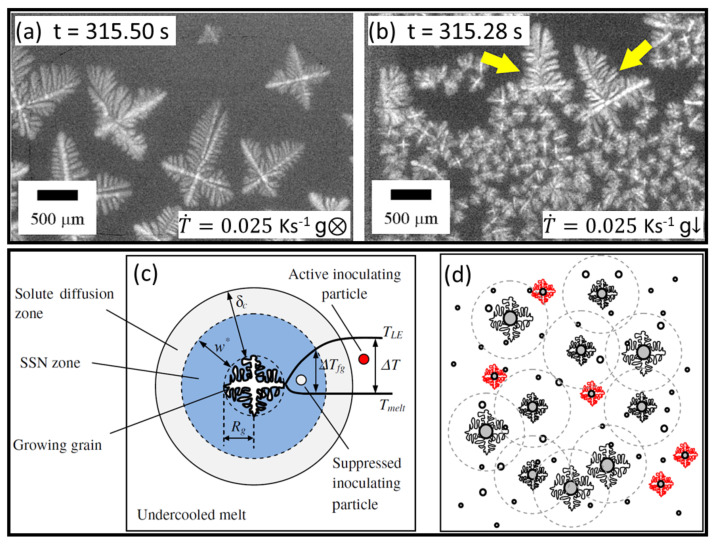
Radiographs of an Al-20wt%Cu alloy inoculated with TiB${}_{2}$, solidifying at 0.025 K s${}^{-1}$ under (**a**) horizontal and (**b**) vertical solidification conditions [64]. Schematic description of solute suppressed nucleation zone (SSNZ) (**c**) around an individual crystal and (**d**) developed by an ensemble of growing crystals [87].

Murphy et al. studied the buoyancy effect on the number density of α-Al dendrite formation events in TiB${}_{2}$-inoculated Al-Cu alloys using laboratory-based radiography apparatus, which allowed for both vertical and horizontal solidification arrangements [64]. For the same cooling rate and at the same time instant during near-isothermal cooling, horizontal solidification (Figure 2a), which removed the gravity effect from the imaging field of view, led to a coarser α-Al grain size and a much smaller grain number density than vertical solidification (Figure 2b). Compared with horizontal solidification where all the grains remained static after formation (Figure 2a), almost all the grains in vertical solidification were mobile and floated upwards as a result of buoyancy (due to the lower density of solid α-Al grains than the surrounding Cu-rich liquid), with only two grains remaining static (indicated by the yellow arrows in Figure 2b), possibly due to nucleation on oxides on the sample wall. The buoyancy-induced grain motion in the vertical arrangement led to early physical impingement between newly formed gains, restricted grain growth, and facilitated further nucleation events. Further, when solidified horizontally, a distinct “nucleation separation distance” could be easily noticed (Figure 2a), within which no further nucleation event was observed, and this distance was inversely proportional to the cooling rate [64].

Although not clearly elucidated in the work, a link can be noticed between the “nucleation separation distance” observed in situ and the well-known solute suppressed nucleation theory [87,88]. During the growth of an α-Al crystal, solute (in this case Cu) is rejected and enriches the local liquid near the solid–liquid interface, so that its equilibrium liquidus decreases, leading to a zone ahead of the solid/liquid interface where the thermodynamic driving force for any further nucleation (undercooling) is temporarily eliminated (Figure 2c). This zone is termed the “solute suppressed nucleation zone” (SSNZ) [10,87], or “nucleation-free zone” (NFZ) [88]. As multiple grains nucleate and grow in the field of view, their solute diffusion fields overlap and suppress subsequent nucleation on nucleant particles within the SSNZ (Figure 2d), leading to the “nucleation separation distance” observed in Figure 2a.

**Figure 3:**
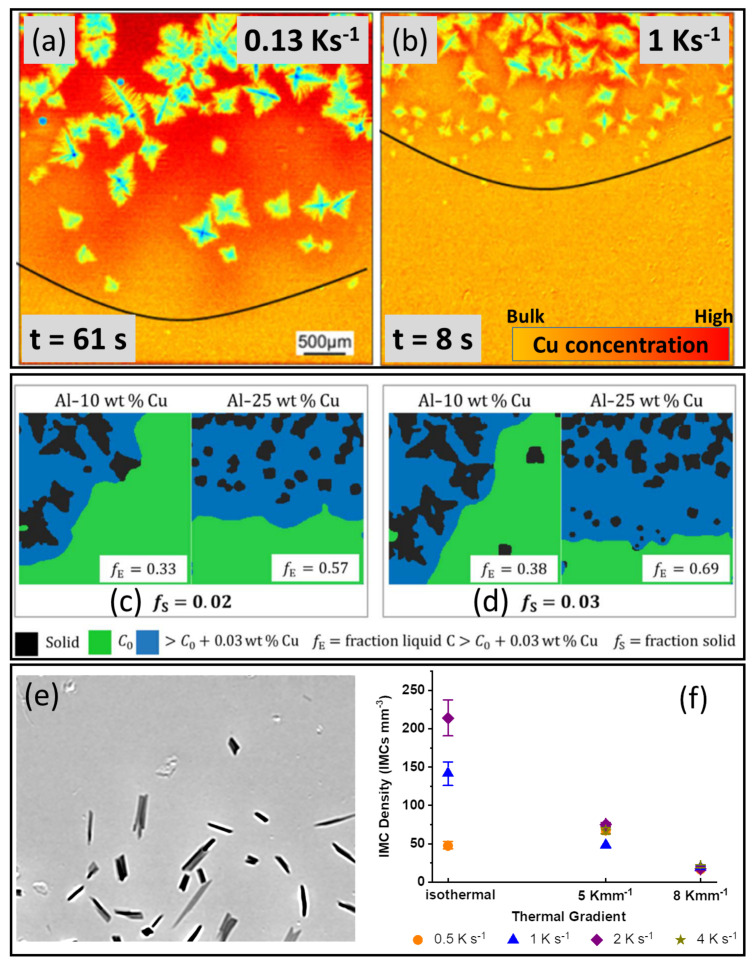
False-coloured radiographs of solidifying Al-15wt%Cu inoculated with TiB${}_{2}$ at (**a**) 0.13 K s${}^{-1}$ and (**b**) 1 K s${}^{-1}$ [12]. Contour maps showing the fraction of Cu-rich liquid ($f_{E}$, coloured in blue) at a solid fraction of (**c**) $f_{S}=0.02$ and (**d**) $f_{S}=0.03$ during solidification of Al-10wt%Cu and Al-25wt%Cu. The Cu-rich liquid was defined as liquid with a Cu concentration 0.03 wt% higher than the bulk concentration [10]. (**e**) A radiograph of primary Al${}_{13}$Fe${}_{4}$ crystals in a solidifying Al-3wt%Fe alloy at 0.5 K s${}^{-1}$ and 5 K mm${}^{-1}$ [15]. (**f**) Final Al${}_{13}$Fe${}_{4}$ number density as a function of thermal gradient in Al-3wt%Fe [15].

A number of in situ studies have been conducted to understand better these types of solute effects on α-Al nucleation [9,10,11,12,13,14]. For example, meaningful trend-wise insights were gained into the influence of solidification conditions such as cooling rate on the size of the SSNZ and the overall grain refinement effect. Figure 3a,b shows false-coloured radiographs of a TiB${}_{2}$-inoculated Al-15wt%Cu alloy solidifying at two cooling rates [12]. The liquid region in the vicinity of the growing α-Al grains was more enriched in Cu. Qualitatively, the same alloy, solidified at the higher cooling rate, had a narrower SSNZ and a reduced spacing between neighbouring nucleation events, contributing to a larger grain number density [12]. The reduced SSNZ at a higher cooling rate was consistent with earlier observations by Murphy et al. [64].

Based on these qualitative insights, Liotti et al. then used a combination of radiography and machine learning to investigate the role of alloy composition, cooling rate and instantaneous local solute concentration in equiaxed α-Al dendrite nucleation in the same alloy system [10]. Using a “reverse time” approach, they measured the instantaneous liquid Cu concentration in a small cluster of image pixels just before a new α-Al dendrite formed at that point. They were able to estimate the undercooling of over 14,000 α-Al grain formation events. Crystals formed in distinct waves rather than semi-continuously, and the waves were more intense for more concentrated alloy compositions. There was no link between the width of the SSNZ and this crystal formation behaviour, but instead it was the overall fraction of Cu-rich liquid in the field of view that dominated the crystal formation behaviour [10]. The solute-enriched liquid fraction increased from 0.33 to 0.57 as the bulk composition increased from 10 to 25 wt% Cu at a solid fraction of 0.02 (Figure 3c), and from 0.38 to 0.69 at a solid fraction of 0.03 (Figure 3d). As a result, in the Al-25wt%Cu alloy, the activity of the more nucleation-efficient TiB${}_{2}$ additions of relatively large diameter—but few in number—was more likely to be solute suppressed. Instead, grain formation occurred on the less efficient (i.e., smaller diameter) but more populous TiB${}_{2}$ particles that were activated eventually as cooling proceeded, leading to distinct grain formation bursts that enhanced the overall efficiency of grain formation [10].

More recently, increasing attention has been given to the formation of minority intermetallic compound (IMC) phases [15,16,26,104], particularly Fe-rich IMCs, which often form as secondary phases in both cast and wrought alloys and which may grow into coarse (up to several millimetres [106]), plate-like morphologies that undermine alloy ductility and toughness [107,108].

Feng et al. investigated the formation of Al${}_{13}$Fe${}_{4}$, one of the most commonly formed Fe-rich IMCs in commercial Al alloys, in a model, hypereutectic Al-3wt%Fe alloy under both isothermal and directional conditions [15]. Figure 3e shows an example radiograph of Al${}_{13}$Fe${}_{4}$ forming as the first, primary phase. By studying approximately 4500 crystal formation events, they showed that additions of TiB${}_{2}$ and TiC inoculants consistently enhanced IMC formation under all solidification conditions. Further, the IMC number density was dominated by the thermal gradient, decreasing sharply from 214 ± 24 IMCs mm${}^{-3}$ under isothermal conditions to 17 ± 3 IMCs mm${}^{-3}$ at the highest thermal gradient of 8 K mm${}^{-1}$ and a cooling rate of 2 K s${}^{-1}$ (Figure 3f). A model for IMC formation was proposed by considering the distribution of the available undercooling in the liquid ahead of a growing IMC. The model was able to describe how the probability of IMC nucleation on a potent nucleant particle was controlled by the size of the undercooled liquid volume and the magnitude of the undercooling, which were in turn dominated by the interplay between the thermal gradient and the local solute field [15]. However, due to limitations in X-ray absorption contrast, detailed quantitative analysis of the solute diffusion fields around growing IMCs and measurement of the associated solute-affected zone were not possible [15].

Therefore, to investigate further solute suppression effects on IMC formation, the nucleation of primary Pt-rich IMC crystals in a model Al-Pt-Er alloy as an analogue of Al${}_{13}$Fe${}_{4}$ was investigated [16]. The micro-segregation of Pt into the primary Pt-rich IMCs provided much stronger absorption contrast between the solid IMCs and the surrounding Pt-depleted liquid and enabled early detection of IMC formation events, and more importantly, the resolution of the developing solute diffusion fields. By considering the interaction between an ensemble of IMCs and potential nucleants, and using time-resolved measurement of the Pt-depleted liquid fraction, which was technically impossible for the earlier Al-Fe [15], the IMC formation model was extended. The probability of IMC nucleation at any instant depended not only on the available liquid undercooling in front of an individual IMC but also on the number of IMCs already formed and the associated liquid fraction that was solute depleted, which in turn depended on the macro-scale cooling rate and thermal gradient conditions. Overall, the formation behaviours of the ordered, Fe-rich and Pt-rich IMCs were shown to be consistent with models originally developed for an α-Al solid solution [10,87,88].

### 3.2. Crystal Growth

**Figure 4:**
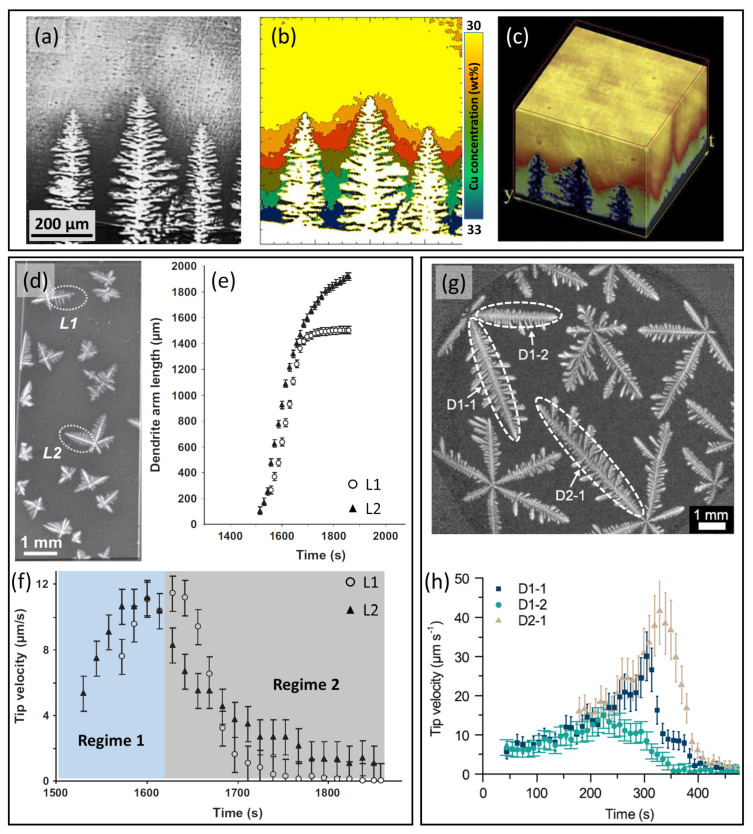
(**a**) A radiograph of growing columnar α-Al dendrites in an Al-30wt%Cu alloy solidifying at 0.6 K s${}^{-1}$ [92]. (**b**) The corresponding Cu-concentration contour map [92]. (**c**) Time-resolved Cu-concentration contours stacked into a 3D volume, with time being the third dimension [92]. (**d**) A radiograph of equiaxed α-Al dendrites growing in an Al-10wt%Cu alloy at 0.008 K s${}^{-1}$ [17]. (**e**) Length evolution of dendritic arms L1 and L2 (as denoted in (**d**)) as a function of time [17]. (**f**) Corresponding tip velocities as a function of time [17]. (**g**) A radiograph of growing α-Al dendrites in an Al-45wt%Ge alloy at 0.02 K s${}^{-1}$ [19]. (**h**) Tip velocities of dendritic arms D1-1, D1-2 and D2-1 as a function of time [19].

Most in situ X-ray studies have focused on crystal growth, which is technically less demanding than crystal formation, and particularly on the growth of the primary α-Al phase [17,18,19,20,21,22,92]. Mathiesen et al. studied the growth of columnar dendrites during directional solidification of an Al-30wt%Cu alloy and extracted spatio-temporally resolved solute distributions in the mushy zone [92]. Figure 4a shows a radiograph of growing columnar α-Al dendrites. The Cu concentration in the liquid at the same time instant was presented as a false-coloured contour map (Figure 4b), which showed increasing Cu-enrichment in the inter-dendritic channels. Time-resolved 2D solute contour maps were then stacked into a 3D volume (Figure 4c) and were used to gain information such as the iso-constitutional surface and the solute undercooling at the dendritic tips as a function of time.

By adding TiB${}_{2}$ to promote equiaxed solidification, Bogno et al. studied the growth of equiaxed α-Al dendrites in Al-10wt%Cu under near-isothermal conditions [17]. Figure 4d presents a radiograph of growing equiaxed α-Al dendrites, and Figure 4e shows the evolution of the length of dendritic arms L1 and L2 (as highlighted by the dashed ellipses in Figure 4d) measured as a function of time. Both L1 and L2 underwent an initial rapid growth, followed by deceleration. On differentiating the dendritic arm length, the tip velocities as a function of time in Figure 4f showed two distinct regimes. The first regime was marked by a steady increase in tip velocity up to a peak of ∼12 μm/s at ∼1630 s. This region represents the early stage of the equiaxed solidification, when the distance between neighbouring crystals was relatively large such that crystals could be considered isolated (no overlap of diffusion fields). In the second region, as the solidification proceeded, tip velocities decayed rapidly towards zero. This was attributed to the impingement of the diffusion fields between neighbouring grains as the solid fraction increased, reducing the driving force for further growth.

The aforementioned experiments were performed in synchrotrons in a vertical arrangement, and a similar study was then conducted by Becker et al. on a model Al-45wt%Ge alloy using a laboratory X-ray apparatus, where the sample was solidified near-isothermally in a horizontal arrangement (Figure 4g) [19]. Despite the differences in the alloy system and solidification conditions, qualitatively similar two-stage growth behaviour was identified in the tip velocity profiles (Figure 4h). Local variations in the peak velocity probably arose from local differences in the solute concentration due to reduced convection in this arrangement [19].

**Figure 5:**
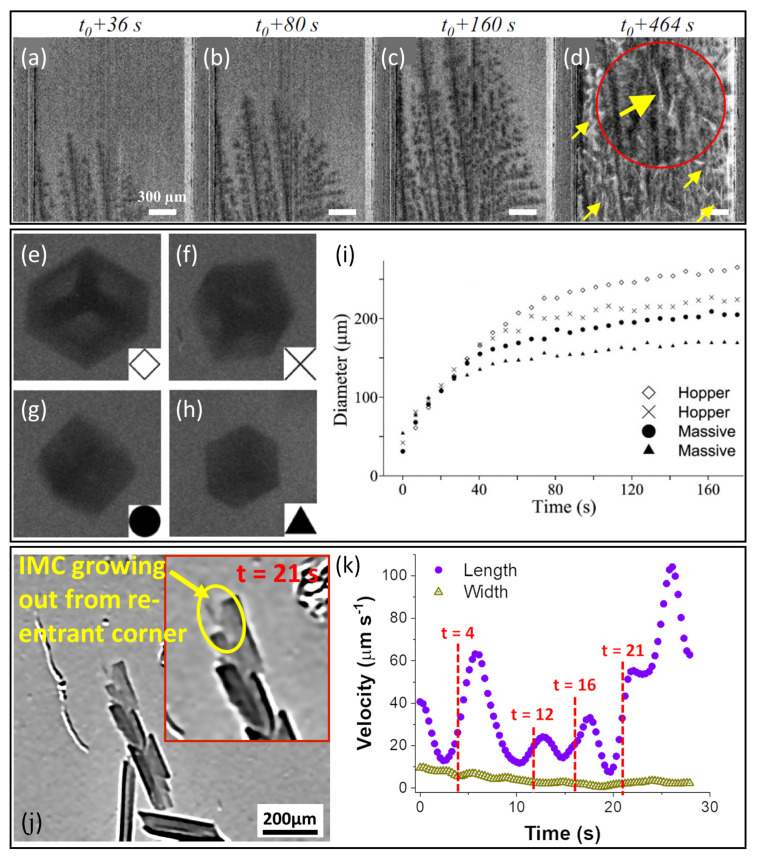
Time-resolved vertical 2D slices from 3D tomographs showing (**a**–**c**) development of columnar α-Al dendrites and (**d**) appearance of secondary β-Al${}_{5}$FeSi IMCs in the inter-dendritic region of an A319 alloy solidifying at 0.1 K s${}^{-1}$ [113]. Time $t_{0}=0$ was set when a dendrite first appeared at the bottom of the field of view. (**e**–**h**) Radiographs of four primary α-Al${}_{15}$(Fe, Mn, Cr)${}_{3}$Si${}_{2}$ crystals formed at 0.5 K s${}^{-1}$ [23]. (**i**) Corresponding size evolution of the four IMCs as a function of time [23]. (**j**) A radiograph of elongated primary Al${}_{13}$Fe${}_{4}$ at 0.5 K s${}^{-1}$, with a magnified inset image showing a thin IMC plate growing out from the re-entrant corner along the original, preferential growth direction [24]. (**k**) Corresponding velocity evolution as a function of time [24].

Unlike the α-Al solid solution phase, which adopts a non-faceted (frequently dendritic) morphology, IMCs are ordered compounds, generally with relatively high entropy of fusion, that grow with a faceted morphology [109,110]. The past decade has seen an increasing number of in situ X-ray studies of faceted IMC growth, spanning growth kinetics [23,24,25,26,60,111,112], volume fraction evolution [113] and morphology analysis [23,102,103,104]. Following earlier proof-of-concept investigations of the growth of secondary Fe-rich IMCs by Wang et al. using X-ray radiography [111] and Puncreobutr et al. using X-ray tomography [60], Cai et al. studied secondary β-Al${}_{5}$FeSi IMCs in an A319 (Al-Si-Cu) alloy using X-ray tomography [113]. Alloy solidification started with the development of columnar α-Al dendrites (Figure 5a–c), followed by the appearance of more X-ray-attenuating (brighter in the tomographs), plate-like secondary Fe-rich IMCs in the inter-dendritic liquid (highlighted by the yellow arrows in Figure 5d). Although the overall evolution of the IMC volume fraction as a function of time was obtained, time-resolved measurement of the growth velocity of individual IMC crystals was technically challenging, principally due to the low volume fraction (<3%) of these IMCs when formed as secondary phases in small liquid pockets towards the later stage of solidification [113].

Alternatively, faceted IMCs have been contrived to form as primary phases in free liquid, to facilitate detection and time-resolved investigation of their growth kinetics. Bjurenstedt et al. studied the growth and morphology of primary α-Al${}_{15}$(Fe, Mn, Cr)${}_{3}$Si${}_{2}$ using laboratory-based X-ray radiography [23]. During near-isothermal cooling at 0.5 K s${}^{-1}$, two types of IMC morphologies were observed from the radiographs showing the cross section of the IMCs perpendicular to the incident X-ray, namely, a hollow hexagon (termed “hopper”, typical of Figure 5e,f) and a solid hexagon (termed “massive”, typical of Figure 5g,h). Time-resolved growth measurement of IMCs in both morphologies showed a nearly monotonic decay of growth velocity with time (Figure 5i). Overall, the hopper and massive crystals had similar average growth velocities of 3.5±0.5μm/s and 2.5±0.6μm/s. Noting that the spatial resolution of the experiments was approximately 5 μm, the velocity measurement might be subject to some error [23].

It has been hypothesised that a twin plane re-entrant (TPRE) mechanism may be involved in the growth of Fe-rich IMCs, based on frequent microscopy observations of growth twins in β-Al${}_{5}$FeSi and Al${}_{13}$Fe${}_{4}$ [110,114], where twinning is believed to facilitate the formation of low-energy sites for relatively easy atom attachment that results in the anisotropic growth of these faceted crystals. Using synchrotron X-ray radiography, frequent microscopic twins were observed by Feng et al. in growing Al${}_{13}$Fe${}_{4}$ crystals that were confirmed by post-solidification electron back-scattered diffraction (EBSD) [24]. A TPRE growth mechanism was identified: the repeated formation of twins during solidification led to the repeated formation of re-entrant corners at twin boundaries that facilitated crystal growth along preferential directions, forming elongated plates. For example, the magnified inset image in Figure 5j highlights a thin IMC plate growing out from a re-entrant corner of Al${}_{13}$Fe${}_{4}$. The time-resolved growth velocity of the crystal showed a periodic fluctuation (Figure 5k), and there was always a peak in the growth velocity following the development of a re-entrant corner (the time instant of which is labelled in Figure 5k). This finding is in good qualitative agreement with the TPRE growth mechanism hypothesised by Adam and Hogan based on post-solidification microscopy of the same phase [110]. More recently, the same growth behaviour has been observed by 3D tomography [25] and by post-solidification EBSD over a wider range of cooling rates [115]. The average growth velocity at 0.5 K s${}^{-1}$, measured along the preferential growth direction of 41 IMCs, was 23±7μm/s [24], which is in a similar range to the 34±20μm/s reported by Wang et al. for β-Al${}_{5}$FeSi at 0.33 K s${}^{-1}$ [111], and both were significantly larger than that reported by Bjurenstedt et al. for α-Al${}_{15}$(Fe, Mn, Cr)${}_{3}$Si${}_{2}$ at 0.5 K s${}^{-1}$ [23]. This difference in growth velocities probably reflected the different crystal symmetries of the monoclinic and cubic IMCs.

### 3.3. Crystal Fragmentation

Section 3.1 mentioned the addition of extrinsic insoluble particles such as TiB${}_{2}$ for refinement of α-Al dendrites and Fe-rich IMCs by promoting their nucleation. Alternatively, grain refinement can be achieved by crystal fragmentation and grain multiplication, typically by the application of external fields such as a pulsed electromagnetic field (PEMF) or ultrasound that act on bulk liquid and solid phases. Induced instability of the crystal growth thermo-solutal environment leads to morphological instability and fragmentation. The full mechanistic understanding of crystal fragmentation is an active area of continuing research interest [27,28,29,30,31,32,116,117,118,119].

**Figure 6:**
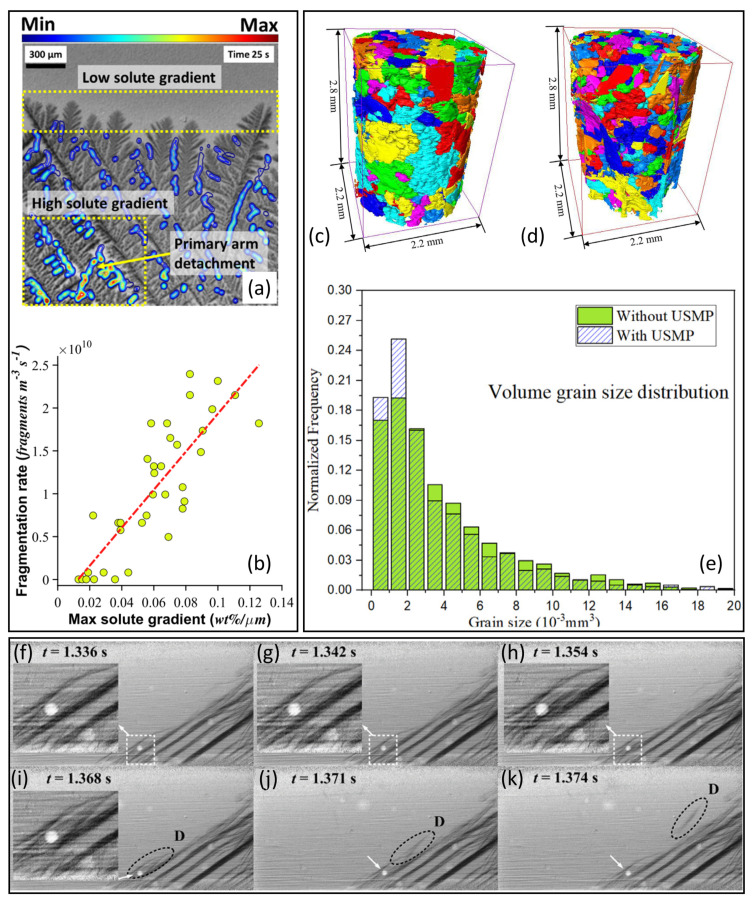
(**a**) Horizontal solute gradient contour map within the inter-dendritic channels of a solidifying Al-25wt%Cu alloy at 1 K s${}^{-1}$ [29]. (**b**) The local dendrite fragmentation rate as a function of the maximum local inter-dendritic solute gradient in the liquid. The red dashed line represents a linear best fit [29]. Rendered tomographs of an Al-15wt%Cu alloy towards the end of solidification (**c**) without and (**d**) with USMP [32]. (**e**) The corresponding α-Al grain size distribution [32]. (**f**–**k**) A radiograph sequence showing the fragmentation of a primary Al${}_{2}$Cu IMC (with the fragment “D” highlighted by a dashed ellipse) [30].

Using synchrotron X-ray radiography, Liotti et al. developed a technique to study the effect of a PEMF on dendrite fragmentation [27], and this technique was then used together with detailed solute gradient measurements (Figure 6a) to establish a link between local changes in liquid composition and the spatial and temporal distribution of dendrite fragmentation events during directional solidification of Al-Cu alloys [29]. The work proved that direct mechanical action by the applied Lorentz force was not the dominating factor for dendrite fragmentation. Instead, the local dendrite fragmentation rate showed an approximately linear relationship with the local solute concentration gradient (Figure 6b). A solute-driven model of dendrite root re-melting was then proposed that related perturbation of the steep liquid solute concentration gradient to local re-melting at vulnerable regions of higher curvature, such as dendrite roots [29].

Zhang et al. studied dendrite fragmentation with and without ultrasound melt processing (USMP) in an Al-15wt%Cu using X-ray tomography [32]. Comparing solidification without (Figure 6c) and with USMP (Figure 6d), 10 s of USMP led to a noticeable reduction in the final grain size (Figure 6e). The enhanced tendency to fragmentation under USMP was attributed predominantly to the re-melting of dendrites induced by increased thermo-solutal perturbation, i.e., a similar mechanism to that proposed by Liotti et al. for fragmentation under PEMF [29].

In comparison, less attention has been paid to the fragmentation of IMCs. Using high-speed X-ray radiography at a frame rate up to 1000 Hz, Wang et al. studied the effect of USMP on the fragmentation of primary Al${}_{2}$Cu in solidifying, hypereutectic Al-35wt%Cu [30]. When USMP was applied to the alloy melt that contained pre-formed coarse, elongated Al${}_{2}$Cu arrays (Figure 6f–h), fragmentation was induced, and fragments detached from the pre-formed IMC array and partially re-melted (a typical example is labelled “D” in Figure 6i–k). Perturbation due to the transport of hot liquid via acoustic streaming flow to the growing IMC array was suggested as the predominant reason accounting for IMC fragmentation [30]. This study demonstrated the possibility of refining primary IMCs by fragmentation in a hypereutectic alloy. It remains to be seen if X-ray imaging can be applied effectively for the study of any fragmentation of IMCs in engineering alloys in which IMCs normally form as low-volume-fraction secondary phases in tortuous inter-dendritic channels.

## 4. Morphological Transition

During solidification, crystals often undergo morphological transitions associated with thermal or solutal perturbation. In situ studies of these transitions have mainly focused on the instability of a planar solid/liquid interface [33,34,37,38], the cellular-to-dendritic transition [35,37,38,39] and the columnar-to-equiaxed transition (CET) [40,120,121,122,123,124].

**Figure 7:**
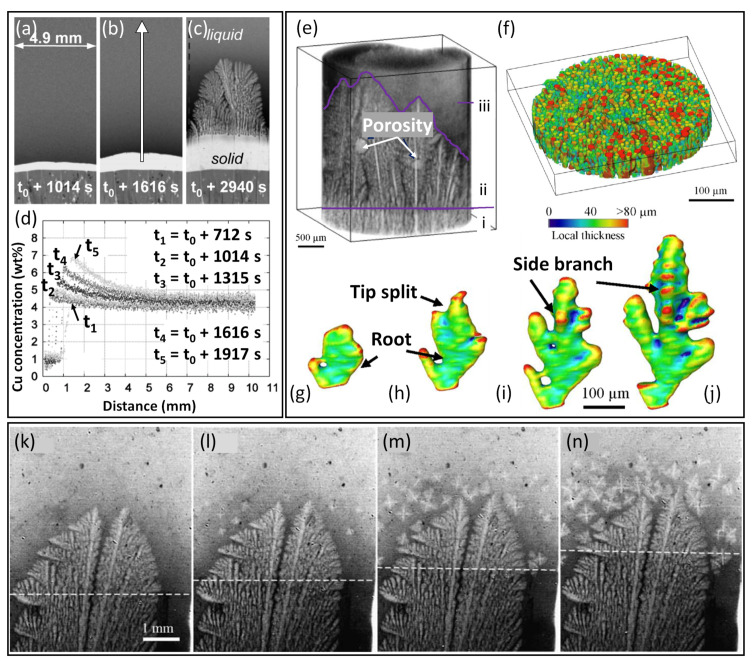
(**a**,**b**) Radiographs showing α-Al growing with a planar interface during the earlier stage of unidirectional solidification of Al-4wt%Cu at 0.008 K s${}^{-1}$ [33]. (**c**) A radiograph showing a dendritic microstructure developed from the original planar interface [33]. (**d**) Solute profiles measured along the white arrow in (**b**) at different time instants during solidification [33]. (**e**) Volume rendering of a solidifying Al-5wt%Cu alloy, showing three distinct zones: cellular (region i), columnar dendritic (region ii) and fully liquid (region iii) [35]. (**f**) The cellular structure near the bottom of the field of view (region i) in (**e**) [35]. (**g**–**j**) An example of an individual cell transitioning to a dendrite [35]. (**k**–**n**) A radiograph sequence showing the CET in an Al-3.5wt%Ni alloy [120].

Buffet et al. and then Bogno et al. investigated solute segregation in front of a planar solid/liquid interface and the resulting instability, during early-stage directional solidification of an Al-4wt%Cu alloy (Figure 7a–c) [33,34]. Figure 7a,b shows radiographs of a planar α-Al solidification front propagating upwards, anti-parallel to the imposed thermal gradient. As explained in Section 3.1, Cu was rejected by the growing α-Al at the solid/liquid interface, enriching the local liquid and gradually diffusing down the concentration gradient with distance from the interface towards the bulk alloy composition (Figure 7d). This local Cu enrichment destabilised the growing planar interface and caused it to break down and transition to a dendritic microtructure (Figure 7c).

Using 4D (3D plus time) tomography, Cai et al. captured the first stage of a cellular-to-dendritic transition in a solidifying Al-5wt%Cu alloy [35]. Figure 7e shows the 3D morphology of the solidifying alloy. Three distinct regions were identified in the tomography field of view, namely, the bottom region (region i in Figure 7e) that was mainly composed of cellular grains, a middle region that comprised columnar dendritic grains (region ii in Figure 7e) and a fully liquid region towards the top of the solidifying sample (region iii in Figure 7e). Figure 7f shows the cellular structure near the bottom of the field of view (region i in Figure 7e), where each cellular grain is coloured according to the local thickness. It was speculated that the cellular structure was developed via epitaxial growth from un-melted solid below the field of view. This cellular growth was then followed by a rapid transition to columnar dendritic growth across the solidification front. Figure 7g–j shows an example transition of an individual α-Al crystal from cellular to dendritic morphology [35].

During the growth of columnar dendritic arrays, a transition from columnar to equiaxed grain morphology occurs when the liquid ahead of the growth front is sufficiently undercooled such that new equiaxed grains nucleate ahead of the growing columnar zone. Understanding the physical mechanisms controlling the CET is critical for industrial applications because, depending on the type of applications, one type of grain morphology is preferred, for example, equiaxed grains in car engines and columnar grains in turbine blades. Some earlier in situ studies of CET mechanisms were carried out by Reinhart et al. during directional solidification of an Al-3.5wt%Ni alloy [120]. First, columnar dendritic growth was contrived by pulling the sample at a relatively low velocity of 1.5 μm/s (corresponding to a lower cooling rate) at a constant thermal gradient of 2 K mm${}^{-1}$ (Figure 7k). Then, to induce a CET, a sharp increase in the pulling velocity to 15 μm/s was applied, following which a band of equiaxed grains started to form in the undercooled melt ahead of the growing columnar dendrites (Figure 7l). Columnar growth was then stopped, and equiaxed grains continued to form and grow in a repeated manner as the solidification proceeded (Figure 7m,n), leading to a stable, equiaxed mushy zone. Importantly, columnar growth largely stopped before actual physical impingement with the newly formed equiaxed grains, which supported the importance of solute field impingement during CET proposed by Martorano et al. [125].

Using the same approach, a CET was also observed in situ in Al-Cu alloys [40,121,122,123]. Ngomesse et al. investigated the CET in an Al-20wt%Cu alloy on Earth and in micro-gravity on board the MASER-14 sounding rocket using X-ray radiography [40]. Unlike the earlier work that reported solutal impingement as the predominant mechanism for CET, this research showed that physical (mechanical) impingement also played a role in inducing CET in both gravity and micro-gravity conditions: shrinkage-induced flow “dragged” the newly formed, free floating equiaxed grains towards the columnar region (i.e., the cooler part of the sample) and blocked the growth of the original columnar front [40]. This observation suggested a significant influence of solidification shrinkage flow, even in the relatively thin ∼200 μm radiography sample.

## 5. Solidification Defects

During casting, microstructural evolution can be accompanied by formation of solidification defects such as porosity and hot tears. These solidification defects can significantly downgrade the final mechanical properties such as tensile ductility and fatigue life. Since the 1990s, in situ X-ray imaging techniques have been used to understand better the formation of solidification defects [43,44,46,126,127,128,129] and their interaction with other microstructural features [41,45,99,130,131,132,133].

### 5.1. Porosity

Using a micro-focus X-ray source and a Bridgman furnace (termed an “X-ray temperature gradient stage”), Lee and Hunt observed the formation of porosity in real time during directional solidification of Al-Cu alloys, and quantified the formation temperature and size evolution of individual pores as a function of solidification velocity, thermal gradient and alloy composition [126]. Their experimental findings suggested that the solidification velocity had the strongest effect on pore formation and growth, and that an increased solidification velocity led to a reduced pore size and an increased pore density. Comparison with predictions from analytical models indicated that pore formation and growth under the solidification conditions studied were mainly driven by the diffusion of hydrogen rather than inadequate liquid feeding. However, once a gas pore was nucleated, shrinkage could help grow pores further [126].

**Figure 8:**
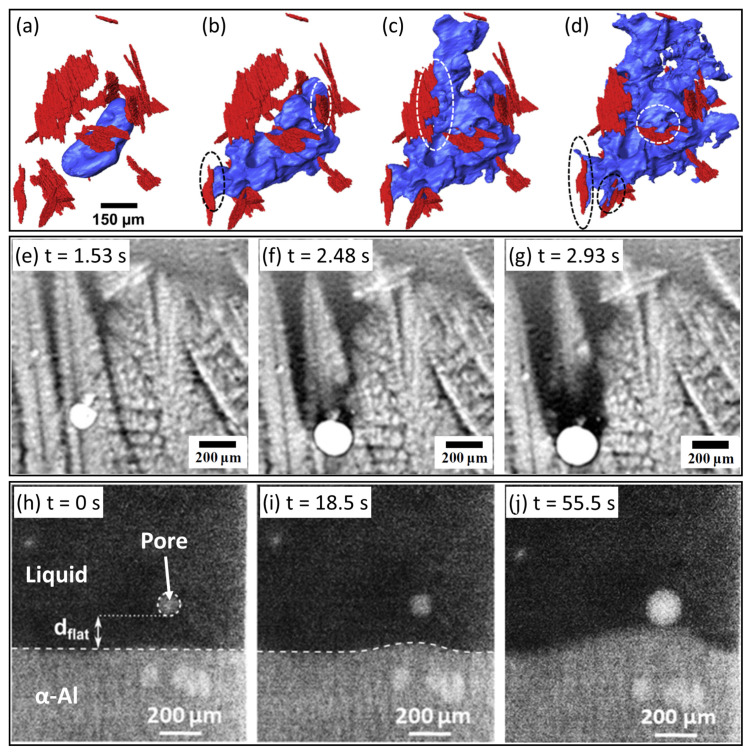
(**a**–**d**) A 3D rendering of a growing pore (blue) in the presence of secondary Fe-rich IMCs (red) in an A319 alloy. The white and black ellipses highlight the contact between the IMCs and the growing pore [41]. (**e**–**g**) A radiograph sequence showing the growth of a pore in the inter-dendritic region that induced dendrite fragmentation, in a directionally solidified Al-12wt%Ge alloy [134]. (**h**–**j**) Radiographs showing a pore growing ahead of a planar α-Al solidification front, causing the front to bulge during directional, horizontal solidification of an Al-10wt%Cu alloy [45].

Following on, Puncreobutr et al. performed a series of tomographic studies on solidification porosity, with a particular focus on the influence of secondary Fe-rich IMCs on pore formation [41,99,130,131]. Although pores did not form directly on the Fe-rich IMCs, the presence of these secondary IMCs tended to block the inter-dendritic channels critical to liquid mobility, and to reduce permeability and increase pore tortuosity. Figure 8a–d shows an example of a pore (rendered in blue) growing in a relatively tortuous part of the space between multiple Fe-rich IMCs (rendered in red) towards the later stage of solidification of an A319 (Al-Si-Cu) alloy. However, due to the limited number of pores in the volume studied and the single experiment performed, a systematic analysis of the pore formation sites and the role of IMCs in pore formation was not possible [41].

Murphy et al. studied the formation and growth of gas porosity in the inter-dendritic region and its effect on the growing dendrites in an Al-12wt%Ge alloy [134]. Porosity led to distortion of the semi-solid mushy zone, and caused remelting and fragmentation of growing columnar dendrites (Figure 8e–g). Pores can form not only in the mushy zone but also in the free liquid. In a recent work by Werner et al., the researchers studied the interaction between gas porosity and a planar solidification front in an Al-10wt%Cu alloy, using a horizontal, laboratory-based set-up [45]. A pore forming ahead of the advancing planar solidification front caused the local front to deform and bulge (Figure 8h–j). This could result from a distortion in the local thermal field as induced by the pore, which was more thermally insulating than the surrounding liquid metal.

### 5.2. Hot Tears

Hot tearing, or hot cracking, is a highly damaging, unrecoverable, large-scale defect that commonly forms during casting of a wide range of Al-, Fe-, Ti- and Ni-based engineering alloys and can be a critical limiting factor of alloy castability [135]. Hot tearing primarily arises from thermally induced tensile strain due to shrinkage associated with the liquid–solid transformation and inadequate/restricted liquid feeding towards the terminal, high-solid-fraction stage of solidification. Once formed, hot tears can continue to propagate and may lead to severe fracture of components during service [135]. So far, in situ X-ray imaging studies of hot tears have mainly relied on semi-solid deformation by uniaxial tension to simulate the thermal tensile stress induced by solidification shrinkage [41,44,127,128,129,133,136].

**Figure 9:**
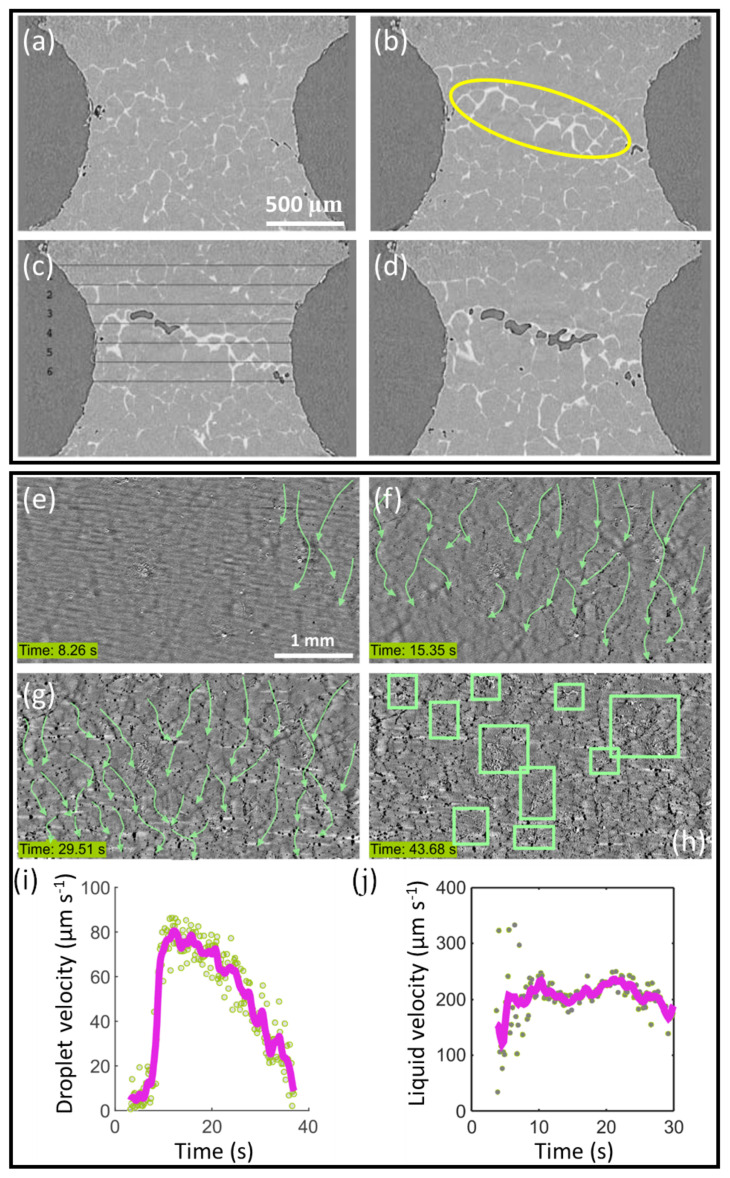
(**a**–**d**) Longitudinal section of a tomograph sequence showing the microstructure of semi-solid Al-8wt%Cu under uniaxial tension [128]. (**e**–**g**) A radiograph sequence of emulsified Pb droplets moving down the inter-dendritic liquid channels between pre-developed equiaxed α-Al dendrites, following paths indicated by the green arrows. Time t=0 s was set when an α-Al dendrite started to form in the field of view. (**h**) A radiograph showing hot tears (indicated by green boxes) formed towards the end of solidification. (**i**) The mean Pb droplet velocity as a function of time. (**j**) The estimated velocity of the liquid flow as a function of time.

Figure 9a–d shows a longitudinal section of a tomograph sequence of Al–8wt%Cu strained at 555 ${}^{\circ }C$ (just above the alloy eutectic point of 548 ${}^{\circ }C$) at a displacement speed of 0.1 μm/s [128,136]. A notch was machined around the middle of the specimen in order to ensure strain localisation in the imaging field of view. In the initial stage of deformation, grains deformed and re-arranged to accommodate the tensile strain, and meantime the inter-granular liquid (the brighter phase within the yellow ellipse in Figure 9b) flowed into the notched region subject to the highest strain. As deformation continued, the inter-granular liquid was no longer able to feed the deformed zone, and cracks formed (Figure 9c,d). The results of the semi-solid tensile test were compared with predictions of a coupled hydro-mechanical granular model proposed by Rappaz et al. [137] and showed good qualitative agreement. The in situ experiment together with the 3D granular modelling suggested that hot tears initiated in the widest inter-granular liquid channels connected with the free (oxidised) surfaces [136].

However, details of how the inter-granular liquid flow rate, the local liquid pressure and crack initiation and growth are inter-related have yet to be fully resolved. This is principally due to the high solid fraction (>80 vol%) and the presence of secondary phases in the inter-granular channels that make imaging and quantification challenging. Nonetheless, Agarwal et al. managed to capture the liquid feeding in inter-granular regions during the terminal stage of solidification of a dual-phase steel [42], and estimated an average liquid flow of 400μm/s–500μm/s, induced by an associated local liquid pressure drop of approximately 10 kPa [42].

Very recently, Liotti et al. developed a technique based on X-ray radiography to investigate the links between inter-dendritic flow behaviour and the onset of hot tearing. Pb was added at 1 wt% to Al to form micron-scale liquid Pb droplets in liquid Al during cooling—effectively a liquid metal emulsion—due to the wide miscibility gap between liquid Al and liquid Pb [138]. The Pb droplets were small enough to follow the liquid flow in the final stages of solidification, and were used as X-ray absorbing tracer particles to quantify shrinkage-induced flow in the inter-dendritic channels, up to the point of hot tear formation. Figure 9e–g presents a radiograph sequence of Pb droplets moving along the inter-dendritic liquid channels between pre-developed equiaxed α-Al dendrites, following paths indicated by the green arrows. Figure 9h shows a radiograph towards the end of solidification in which the green boxes highlight the location of hot tears (brighter). By tracking many hundreds of individual Pb droplets simultaneously frame by frame, the mean droplet velocity as a function of time was determined, as shown in Figure 9i. The velocity of the liquid alloy flow (Figure 9j) was then estimated from the droplet velocity following procedures described in [139,140]. Finally, liquid flow was related to local pressure changes and a critical pressure for hot tear formation was identified. Overall the sequence presented in Figure 9e–g showed a median liquid flow of ∼200 μm/s and a typical pressure drop of ∼30 kPa; both were of the same order of magnitude as those suggested in [42].

## 6. Non-Equilibrium Metal Processing

Additive manufacturing (AM) techniques such as laser powder bed fusion (LPBF) and directed energy deposition (DED) have been increasingly explored for the manufacture of products for the aerospace, automotive and biomedical sectors, because of their capability to produce complex, near-net-shape components directly from a 3D CAD file [3,141]. In situ X-ray imaging with high temporal resolution (microseconds) provided by synchrotron X-ray sources is being used to investigate the process–structure relationships in these processes, such as the high speed melt pool behaviour, heat source–powder bed interactions and defect formation [48,49,50,51,52,53,54]. It is also being used to shed light on other rapid solidification processes such as laser spot welding [68,142,143].

### 6.1. In Situ X-ray Imaging of Rapid Solidification

**Figure 10:**
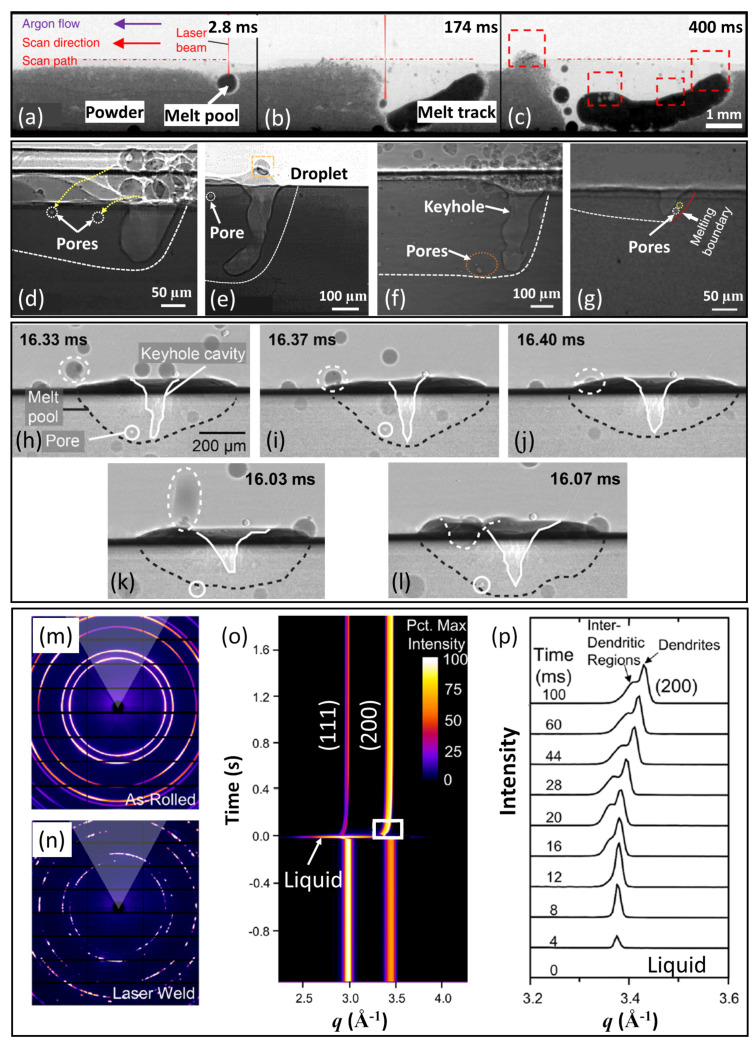
(**a**–**c**) A radiograph sequence showing the evolution of the melt track morphology at the onset, intermediate and final stages of LPBF, respectively [50]. (**d**) A radiograph showing the transfer of pores from the powder bed into the melt pool [147]. (**e**) Pore formation due to surface fluctuation of the melt pool [147]. (**f**) Pore formation due to collapse of the keyhole [147]. (**g**) Pore formation along the melting boundary [147]. Radiograph sequences showing the interaction between powder particles and the melt pool during DED process for (**h**–**j**) a particle with a lower velocity estimated at ∼1m/s and (**k**,**l**) a particle with a higher velocity estimated at ∼10m/s [54]. Debye–Scherrer rings of a Mn-Fe-Co-Ni-Cu high-entropy alloy (**m**) in a cold-rolled state and (**n**) after laser welding [68]. (**o**) Time-resolved 1D diffraction pattern as a function of the reciprocal lattice vector *q*. Time t=0 indicates the onset of solidification [68]. (**p**) Evolution of the (200) peak at a selected time interval (indicated by a box in (**o**)) [68].

Using spatio-temporal resolutions of 6.6 μm and 196 μs, Leung et al. imaged the melt track morphology as a function of laser power and scan speed during LPBF of a Fe-Ni alloy [50]. The radiograph sequence in Figure 10a–c shows the evolution of the melt track morphology at the onset, intermediate and final stages of LPBF, respectively. After melt pool formation (Figure 10a), the melt pool evolved into an elongated melt track that followed the laser beam surface interaction and extended simultaneously towards the bottom of the powder bed as the scan proceeded (Figure 10b). The melt track then solidified and deformed upwards as the laser moved away (Figure 10c). Later, similar investigations of melt pool geometry as a function of processing parameters were also performed by other researchers using the same technique [144,145,146].

Apart from the melt pool behaviour, studies have also focused on defect formation during LPBF, such as porosity and hot tearing. A number of pore formation and evolution mechanisms have been proposed. In the aforementioned work, Leung et al. imaged pores formed in the melt pool and proposed three types of pore evolution: pore coalescence, dissolution and dispersion into smaller pores [50]. Later, Hojjatzadeh et al. carried out a detailed investigation into pore formation during LPBF and identified primarily four mechanisms: transfer of pores from the powder bed into the melt pool (Figure 10d), pore trapping by surface fluctuation (e.g., protrusion or droplet) of the melt pool (Figure 10e), pore formation induced by keyhole (Figure 10f) and pore formation on the melting boundary (i.e., the solid/liquid interface) (Figure 10g) [147]. At high energy density (high laser power and/or low scan speed) in particular, the melt pool became relatively deep and narrow, and could easily become unstable, with pores formed as the beam translated (Figure 10f). In a study dedicated to the dynamics of so-called “keyhole-induced” porosity, pores formed at laser turning points during scanning [51]. This was attributed to the deceleration and re-acceleration of the scan at the beam turning points that caused destabilisation and collapse of the keyhole, trapping gas in the solidifying melt.

As in nearer-equilibrium solidification, hot tearing is also commonly seen in AM of engineering alloys with relatively wide freezing ranges, such as the commercial aluminium alloy AA6061 [148]. A high thermal gradient arising from the scanning heat source in AM results in highly directional columnar grains that are vulnerable to hot tearing. Once formed, hot tears can run the entire length of columnar grains, and may even propagate across multiple layers of the print [148]. So far, in situ studies of hot tearing have mainly focused on AA6061, given its high susceptibility [52,143,149], with a strong link proposed between hot tears and pores. On the one hand, pores acted as preferential sites for hot tear initiation, for example, in the cases of both large keyhole-induced pores and smaller trapped-gas-induced pores [52,149]. On the other hand, once cracks were present in the material, gas trapped in the melt pool could nucleate as pores on the crack surfaces [52]. The strong interaction between pores and hot tears suggested that strategies for their avoidance should be considered in a conjugated way during future material-process design. For example, it was suggested that when tailoring alloy composition for hot tearing resistance in LPBF, consideration should also be given to any resulting effects on the laser absorption behaviour that was linked to keyhole pore formation [52].

In comparison with LPBF where the powder feedstock is in the form of a loosely packed powder bed, in DED, powder is deposited locally from a feed nozzle, directly into a melt pool created by a point heat source such as a laser [150]. Since DED does not rely on a powder bed, it provides a higher degree of geometrical freedom than LPBF and is not subject to build size restrictions. However, the mechanical complexity of the DED set-up makes it challenging to study interactions between the heat source, powder particles and melt pool in situ. Wolff et al. pioneered in situ work by developing a gravity-fed, low-powder-mass flow deposition system that allowed imaging of interactions between individual powder particles and the melt pool [150]. Most powder particles, as they fell under gravity, were scattered away from the melt pool due to a vapour plasma plume induced by the scanning laser. This suggested that an inert carrier gas would be necessary to deliver powder flow into the melt pool. By introducing Ar as a carrier gas, the powder–melt pool interaction for powder particles at different velocities was investigated [54]. Powder particles at a lower velocity (indicated by a white dashed circle in Figure 10h–j) were incorporated into the melt pool by landing onto the melt pool surface, wetting the surface, and then melting. Powder particles at a higher velocity (Figure 10k–l) penetrated the melt pool surface and were incorporated into the melt pool almost instantaneously [54].

Unlike LPBF and DED, which are characterised by a scanning heat source, AM by binder jetting uses an iterative ink-jet printing method, where alternating layers of build material and binding material are deposited to build up components [55,56]. Parab et al. investigated this process in situ using a commercial binder jetting system with a droplet-on-demand ink-jet print-head. Significant drift of ink-jet binder droplets was observed, and the impact of binder droplets on the previously deposited powder bed led to movement and ejection of a large volume of powder particles, leaving a zone depleted of build material [55]. These insights related less to the simultaneous heat, composition and mass flows typical of solidification imaging studies, but nonetheless provided valuable understanding that aided process development and optimisation.

Efforts have been made recently to explore the feasibility of using recycled metal powder in AM processes, to improve their sustainability. For example, meaningful information was extracted by X-ray tomography relating to the recycled powder shape, size distribution and porosity [151,152]. Although these studies were not time-resolved, they suggest that in situ X-ray imaging may play a useful role in developing new AM processes for recycled powder feedstock.

### 6.2. In Situ X-ray Diffraction of Rapid Solidification

In addition to imaging, in situ X-ray diffraction has also been used to understand rapid, non-equilibrium solidification processes, particularly regarding phase transformations in multi-element alloys. Schneiderman et al. studied liquid–solid phase transformations during laser spot welding of a cold-rolled Mn-Fe-Co-Ni-Cu high-entropy alloy [68]. Figure 10m,n shows the Debye–Scherrer rings before (in the cold-rolled state) and after laser welding, respectively. Reduced continuity in the ring pattern can be seen after laser re-melting, due to an increase in the grain size and a preferential grain orientation in the laser melted zone, later confirmed by post-solidification electron back-scattered diffraction. Figure 10o shows the time-resolved, integrated 1D diffraction pattern as a function of the reciprocal lattice vector q=4πsinθ/λ, where θ is the Bragg angle and λ is the wavelength of the incident X-ray. Time t=0 indicates the onset of solidification. Figure 10p provides a detailed view (for the region indicated by a white box in Figure 10o) of the evolution of the (200) peak as solidification proceeded. A single, sharp (200) peak first emerged from the amorphous liquid background at t=4 ms, and the peak remained sharp and symmetric for the first 12 ms. At t=16 ms, a broad shoulder started to develop on the left of the original (200) peak, indicating the formation of another phase with a slightly larger lattice parameter. The diffraction pattern remained largely unchanged after 100 ms, which marked the end of solidification. Later, post-solidification microscopy confirmed the formation of two phases, namely, primary dendrites corresponding to the sharp, main peak and eutectics corresponding to the shoulder [68].

Using in situ X-ray diffraction, Zhao et al. revealed the solidification sequence of Ti-6wt%Al-4wt%V during LAM [65]. By examining the evolution of the intensities of the BCC (110) peak and the HCP(101) peak, BCC β-Ti was identified as the first solid phase formed, followed by a rapid solid-state transformation at an estimated rate of >$10^{4}$μm/s, suggesting a possible martensitic transformation from BCC β-Ti to HCP $\alpha^{\prime }$-Ti [65]. More studies were then conducted on this alloy system using the same techniques, revealing insights into phase transformation temperatures and residual strains [66,67]. It is also worth noting that in situ X-ray diffraction continues to extend its application, for example, in the investigation of other non-equilibrium processes such as electromagnetic-assisted sintering [153].

## 7. Conclusions and Outlook

The rapid evolution of in situ X-ray imaging capability, especially improvements in spatial and temporal resolution and the availability of more beamlines, has opened up new applications in solidification science, and contributed to the understanding and development of new solidification processes. Contributions include improved understanding of:

1. The nucleation and formation behaviour of both α-Al and Al-based IMC crystals, including how crystal number density, nucleation rate and nucleation undercooling are affected by solidification conditions and solute suppression effects.
2. The important role of the dynamics of solute redistribution in microstructural instability, such as dendrite fragmentation and various morphological transitions.
3. The growth mechanism of faceted Fe-rich IMCs, where the repeated formation of twin plane re-entrant corners facilitates anisotropic crystal growth and results in IMC crystals of a high aspect ratio.
4. The formation of hot tears by probing liquid flow behaviour and estimating associated liquid pressure drops in the final stages of solidification.
5. The solidification conditions in faster, non-equilibrium processes, including the influence of processing conditions on microstructure and defect formation during AM, and the ever-increasing applications in the development of new processes.

Further, new opportunities for applying X-ray imaging to solidification research will emerge as synchrotrons around the world continue to undergo periodic upgrades [154,155]. For example, a dual imaging and diffraction (DIAD) beamline was commissioned recently at the Diamond Light Source, UK. The dual beam design allows simultaneous collection of images and diffraction patterns from the same region, intended, for example, to identify metastable and impurity phases in a solidifying melt pool during AM [156]. Meanwhile, the development of laboratory-based, micro-focus X-ray sources also continues to progress and provides an increasingly capable and convenient alternative to synchrotron-based imaging, together with more flexibility in the experimental set-up [157,158].

In general, because of the increase in synchrotron X-ray beam flux, data speeds, detector resolution and field of view, further challenges will emerge in data collection, transfer, storage and analysis. In the case where systematic, quantitative information is to be extracted from each of tens of thousands of images, more powerful computers, automated data processing and information extraction algorithms based on machine leaning techniques and artificial intelligence will play an increasingly important role [10,22]. Indeed, these approaches will become the only ways in which the vast imaging and diffraction data sets—increasingly obtained simultaneously—can be quantified, and robust physical insights obtained.

## Data Availability

Not applicable.
